# Feeding Performance of Argentine Stem Weevil Is Reduced by Peramine from Perennial Ryegrass Infected with Endophyte Fungus

**DOI:** 10.3390/insects15060410

**Published:** 2024-06-03

**Authors:** Manuel Chacón-Fuentes, Daniel Martínez-Cisterna, Waleska Vera, Fernando Ortega-Klose, Claudio Reyes, Ignacio Matamala, Andrés Quiroz, Leonardo Bardehle

**Affiliations:** 1 Agriaquaculture Nutritional Genomic Center, CGNA, Temuco 4780000, Chile; manuel.chacon@cgna.cl; 2 Laboratorio de Química Ecológica, Departamento de Ciencias Químicas y Recursos Naturales, Universidad de La Frontera, Av. Francisco Salazar 01145, Casilla 54-D, Temuco 4811230, Chile; 3 Centro de Investigación Biotecnológica Aplicada al Medio Ambiente (CIBAMA), Universidad de La Frontera, Av. Francisco Salazar 01145, Casilla 54-D, Temuco 4811230, Chile; 4 Programa de Doctorado en Ciencias de Recursos Naturales, Universidad de La Frontera, Temuco 4811230, Chile; 5 Escuela de Química y Farmacia, Facultad de Farmacia, Universidad de Valparaíso, Valparaíso 2340000, Chile; 6 Centro de Investigación, Desarrollo e Innovación de Productos Bioactivos, CInBIO, Facultad de Farmacia, Universidad de Valparaíso, Av. Gran Bretaña 1095, Valparaíso 2340000, Chile; 7 Instituto de Investigaciones Agropecuarias, INIA Carillanca, km 10 Camino Cajón-Vilcún, s/n, P.O. Box 58-D, Temuco 4780000, Chile; 8 Carrera de Biotecnología, Universidad de La Frontera, Temuco 4811230, Chile; 9 Departamento de Producción Agropecuaria, Facultad de Ciencias Agropecuarias y Medioambiente, Universidad de La Frontera, Av. Francisco Salazar 01145, Casilla 54-D, Temuco 4811230, Chile

**Keywords:** Argentine stem weevil, *Lolium perenne*, endophyte, alkaloidal extract, peramine

## Abstract

**Simple Summary:**

This study investigates the impact of endophyte-infected ryegrass and the alkaloids (such as peramine) which it produces, on the Argentine stem weevil (*Listronotus bonariensis*), a pest affecting pastures crucial for extensive agriculture. Seven unnamed lines (LE161-LE167), and two *Lolium perenne* cultivars, Jumbo and Alto AR1, were evaluated. Leaves from LE164, LE166, and ALTO AR1 led to significant weight reduction in *L. bonariensis* (−13.3%, −17.1%, and −18.2%, respectively). The corresponding alkaloidal extracts from these lines, grown in greenhouse trials, exhibited an antifeedant effect in laboratory assays, resulting in reduced weevil weight (−12.5%, 8.8%, and 4.9%, respectively). When incorporated into an artificial diet, peramine induced an antifeedant effect. Liquid chromatography analysis revealed the presence of peramine in LE164, LE166, and ALTO AR1 (ranging from 46.5 to 184.2 ng g^−1^), while it was absent in Jumbo and other lines. These findings suggest that endophyte-induced peramine production in specific ryegrass lines negatively impacts the feeding performance of *L. bonariensis*, presenting a potential ecological approach for pest control in pastures crucial for extensive agriculture.

**Abstract:**

One of the primary supports for extensive agriculture is pasture, which can suffer severe damage from insects including the Argentine stem weevil, *Listronotus bonariensis*. The main control method has been the infection of ryegrass with an endophyte fungus, forming a symbiotic association that produces alkaloids. In this study, we evaluated the impact of endophyte and peramine production on the weight of *L. bonariensis* across seven unnamed lines (LE161-LE167), and two *Lolium perenne* cultivars: Jumbo and Alto AR1. *L. bonariensis* adults fed on leaves from LE164, LE166, and ALTO AR1 showed weight losses of 13.3%, 17.1% and 18.2%, respectively. Similarly, the corresponding alkaloidal extract from LE164, LE166, and ALTO AR1 exhibited an antifeedant effect on *L. bonariensis* adults in laboratory assays, as observed through weight loss or low weight gain (−12.5%, 8.8% and 4.9%, respectively). Furthermore, one alkaloid, peramine, also elicited an antifeedant effect when incorporated into an artificial diet. Liquid chromatographic analysis of the alkaloid extract revealed that peramine was present in LE164, LE166 and ALTO AR1 in amounts ranging from 46.5–184.2 ng/g. Peramine was not detected in Jumbo and the remaining experimental lines. These data suggest that *L. bonariensis* were susceptible to peramine produced from endophyte infection in experimental lines LE164 and LE166, as well as ALTO AR1, affecting their feeding behavior.

## 1. Introduction

Pastures play a crucial role globally, providing various ecosystem services and sustaining meat and milk production [1]. Therefore, one of the most critical factors influencing beef and dairy production in Chile and worldwide is the careful selection of cultivars, or combinations of both forage grasses and legumes, to ensure high yields and pasture quality related to nutritional values for animals. In this regard, perennial ryegrass (*Lolium perenne* L.) holds significant standing as it is one of the most commonly cultivated species for both farming and livestock forage production [2]. Several studies have highlighted *L. perenne* as an ideal forage grass due to its adequate seed production, grazing tolerance, good digestibility, and adaptability to various habitats [3,4]. Nonetheless, Lamelas-López et al. [5] have indicated that various groups of invertebrates pose a threat to crops, with arthropods being a primary global menace to agroecosystems due to direct impacts and their potential to transmit diseases to plants. 

The Argentine stem weevil, *Listronotus bonariensis* (Kushel), a native South American species, is a significant economic pest of agricultural grasses. Adult weevils feed on the leaves, leaving characteristic ‘window-like’ feeding scars on the adaxial leaf surface. Eggs are laid in the pseudostem; the larvae bore into the stem and tunnel through the center of tillers, causing substantial damage that can lead to plant mortality [6,7]. In Chile, the first recorded incidence of *L. bonariensis* was reported by Norambuena and Gerding [8] on wheat and barley in the southern region of the country, with a detection of 27.9% and 7.1% attacked axes, respectively. Subsequently, Cisternas [9] reported a 44.4% loss in dry matter production in *L. multiflorum* (biennial ryegrass) due to larval attacks in Osorno, Región de Los Lagos [10]. Several methodologies have been studied to control this insect, including biological, cultural, and chemical methods. In terms of biological control, a study by Shields et al. [11] found *Mictoctonus hyperodae* Loan (Hymenoptera: Braconidae) to reduce weevil feeding by 16.3%. Chemical control relies on the application of pesticides from various chemical families, such as neonicotinoids, pyrethroids, and phosphates. However, none of the evaluated insecticides have proven completely successful in controlling this curculionid due to the larvae’s habits and the high level of adult flight [12,13]. Moreover, the use of these synthetic products has been linked to environmental degradation, the development of resistance, and chemical residues [1,8,9]. Hence, there is global interest in utilizing environmentally friendly alternative pest control methods, such as fungal endophytes (*Epichloë/Neotyphodium* spp.), capable of forming symbiotic associations with *L. perenne* [14]. These endophytic fungi have been reported to confer a range of beneficial attributes to their host grasses, primarily protecting them from mammalian and invertebrate herbivory through the production of secondary metabolites, particularly alkaloids, in exchange for a protected niche and nutrition from the host plant [15,16]. Alkaloids are known for their toxic effects, as they can block (a) neuroreceptors, (b) neuronal signal transduction intermediaries, and (c) ion channels of vertebrates and insects due to their chemical similarity to molecules involved in the transmission of nervous system signals [17]. In the last 30 years, researchers have identified four categories of alkaloids associated with the anti-feeding impact on insects: indole diterpenes (e.g., lolitrem B and epoxyjanthitrems); ergot alkaloids (e.g., ergovaline); pyrrolopyrazines (e.g., peramine); and pyrrolizidines (e.g., lolines). However, only grasses harboring endophytes that putatively produce peramine (i.e., anti-chewing defense) and lolines (i.e., anti-aphid and chewing insect defense) are desirable in pastures because they confer insect resistance without affecting grazing mammals. On the other hand, ergovaline and the indole diterpene, lolitrem B, serve a similar protective function against pasture pests [18,19], but these compounds are toxic to grazing livestock, leading to the development of “ryegrass staggers” syndrome. The consequences of livestock exposure to these compounds include reduced feed intake, vasoconstriction, and reproductive issues [20,21]. Consequently, endophytes have been screened to produce fewer toxic alkaloids for livestock while maintaining the production of insect deterrent alkaloids like peramine [22]. Recently, Parra et al. [10] investigated the incidence of *L. bonariensis* in ryegrass pastures in southern Chile, reporting a significant increase (>30%) in the population and occurrence of this curculionid in recent years. Consequently, there is a crucial need to search for new experimental lines of ryegrass with endophytic fungi to minimize herbivorous attacks. Therefore, the aim of this study was to evaluate the effects of new experimental ryegrass lines hosting endophytic fungi that produce varying amounts of peramine, impacting the feeding performance of *L. bonariensis*. The data generated could significantly benefit plant breeders interested in developing potential new commercial grass cultivars to enhance pasture grasses and reduce *L. bonariensis* damage.

## 2. Materials and Methods

### 2.1. Plant Material

Seeds of the commercial cultivars ALTO AR1, an endophyte-infected ryegrass cultivar (E+), and JUMBO, an endophyte-free cultivar (E−), were obtained from the Agricultural Experimental Station at Carillanca (Temuco, Chile) and served as controls. Additionally, seed from seven experimental lines of *L. perenne* (LE161, LE162, LE163, LE164, LE165, LE166, and LE167) were sourced from the Departamento de Producción Agropecuaria, Facultad de Ciencias Agropecuarias y Medioambiente, Universidad de La Frontera (Temuco, Chile). All these seeds were lines previously infected by the respective institutions. The seeds were germinated on moist filter paper and cultivated under natural conditions for five weeks. Subsequently, *L. perenne* seedlings were transplanted into plant pots (8 cm deep, 5 cm in diameter) filled with previously prepared soil at temperatures exceeding 25 °C during the day. The plants were not fertilized but received daily irrigation with distilled water. For each cultivar and experimental line, 15 pots were set up, each with 10 plants (n = 135). All plants were harvested (the tops were cut near the soil line) at three months.

### 2.2. Evaluation of Endophytes in Plants of L. perenne

The endophyte infection levels of each experimental line and cultivar were determined using the method outlined by Dombrowski et al. [23] before initiating the experiments. Briefly, 40 tillers per cultivar or experimental lines were cut, and the inner epidermis of a leaf sheath was peeled off and placed on a glass slide. Tillers were harvested across all pots sowed per cultivar or experimental lines. Two drops of rose bengal stain (Merck KGaA, Darmstadt, Germany) were added to each sample. After 1–2 min, the samples were covered with a cover slip. Subsequently, the presence or absence of typical fungal mycelium was assessed through microscopic examination (40×), and the infection percentage was calculated. Positive infection of the endophyte was attributed to the presence of the fungus hyphae in the assessed tillers. Three replicates were conducted for each experimental line and cultivar (n = 120).

### 2.3. Isolation and Characterization of Endophytic Fungus in Lolium perenne

For its isolation, the disinfection of *L. perenne* seeds was carried out using 3% sodium hypochlorite, followed by washing with sterile distilled water. Subsequently, the seeds were placed on potato dextrose agar (PDA) supplemented with penicillin and streptomycin (100 µg g^−1^) for 3 weeks at 23 °C. The determination of *Neotyphodium* sp. in *L. perenne* seeds was confirmed through molecular techniques using the methodology proposed by Dombrowski et al. [23]. To obtain the fungus DNA, fungal tissue was taken from petri dishes with strains cultivated from *L. perenne* seeds, which was macerated under sterile conditions with liquid nitrogen. The obtained macerate was processed using the NucleoSpin Soil DNA isolation kit (Macherey-Nagel). During the DNA extraction process, non-denatured DNA was obtained, and the purity index values were analyzed using Gen5. Finally, a PCR reaction was conducted, associating the obtained DNA with the identity of the *Neotyphodium* sp. fungus.

### 2.4. Alkaloid Extraction Procedure and Analysis

Twenty leaves of the *L. perenne* experimental lines and cultivars were sampled at 3 months of age across the pots, following the methodologies outlined by Roylance et al. [24]. In summary, each sample was dried and ground, then 500 mg was extracted with 3 mL of methanol-chloroform (1:1, *v*:*v*) (Merck KGaA, Darmstadt, Germany) for 30 min at room temperature. Subsequently, 3 mL of hexane-water (1:1, *v*:*v*) (Merck KGaA, Darmstadt, Germany) was added, and the samples (6 mL in total) were centrifuged at 1300 r.p.m. Two phases were obtained in each sample (organic and aqueous), with the organic phase being discarded. One milliliter of the aqueous phase was passed through a 100 mg CCX extraction column (UCT 2731 Bartram Road, Bristol, PA, USA) and discarded. Subsequently, 1 mL of HPLC water was passed through the column and also discarded. Finally, alkaloids were eluted with 0.5 mL of 5% aqueous formic acid, collected, and stored in amber vials (1.5 mL) for subsequent high-performance liquid chromatography with diode array detection (HPLC-DAD) analysis. Then, each extract of *L. perenne* underwent filtration through a 0.22 μm membrane, and 20 μL of the filtered solution was injected into a Shimadzu HPLC (LC-20A Prominence, Kyoto, Japan) equipped with a C-18 column (300 × 4.6 mm I.D.; particle size 5 mm) maintained at 30 °C. The analysis was conducted using the methodology proposed by Parra et al. [25]. An isocratic mobile phase comprising 80% guanidinium buffer (A) and 20% acetonitrile (B) was employed at a flow rate of 1 mL/min. The guanidinium buffer was prepared by dissolving 1.44 g of guanidinium carbonate in 800 mL of HPLC water, supplemented with formic acid (1.3 mL/L), and 200 mL of acetonitrile (Merck KGaA, Darmstadt, Germany). Peramine (predominant compound) in extract was monitored at 280 nm, and its identification was established based on the peak retention time compared to a commercial standard (obtained from AgResearch, Hamilton, New Zealand). For the construction of calibration curves, the standard was dissolved in methanol at a concentration of 1000 mg/L. The stock solution was then used to prepare a series of concentrations ranging from 0.01 to 100 mg/L.

### 2.5. Insects

Recently emerged *L. bonariensis* adults were manually collected from an experimental field at the Agricultural Experimental Station at Remehue (Osorno, Chile) between December 2022 and February 2023. Briefly, the adult individuals of *L. bonariensis* were collected freshly emerged from the experimental field. To ensure this, marked areas were established in the field where damage was observed on the stem of the cultivar, indicating the presence of the insect. Subsequently, pupae were collected at ground level. Pupae were carefully collected with soft forceps and observed in the field for one week until emergence. The adults were then transferred to the laboratory and tested. All adults used emerged in the same week. Upon transfer to the laboratory, the individuals were kept in petri dishes under controlled conditions (18–22 °C and an 18L:6D light regimen) on ryegrass devoid of endophytes (E−). Insects used in the bioassays were collected in the same week and were each utilized only once. Before each feeding assay, each L. bonariensis adult was observed for 10 min, 2 h in advance. Only insects actively moving during the experimental period were selected for the no-choice bioassays.

### 2.6. No-Choice Bioassay

An individual adult of *L. bonariensis*, previously weighed (Wi = initial weight), was placed in a Petri dish (94 mm in diameter by 16 mm high) with a leaf (12–15 cm of length) from either the experimental lines or the commercial cultivars (n = 15 for each cultivar and experimental line), positioned in the center (leaves were harvested and used exclusively for this experiment). The insects were allowed to feed for 4 days under dark conditions at room temperature (25 °C ± 2 °C). Subsequently, the adults were weighed again (Wf = final weight) at the end of the bioassays. The feeding performance was evaluated based on the weight gain (Wg), calculated as follows: Wg (%) = (Wf − Wi) × 100/Wi [26]. The commercial cultivars Jumbo (E−) and Alto AR1 (E+) were used as controls.

### 2.7. Alkaloidal Extract Bioassays

10 µL of alkaloid extracts (a new solution prepared using the same methodology described above, only employing water as the solvent) obtained from the experimental lines and cultivars, were individually added to a leaf of the Jumbo cultivar (E−), which is endophyte free, and placed inside a Petri dish (94 mm in diameter by 16 mm high). The leaf was air-dried for 30 s to facilitate solvent evaporation. Subsequently, an individual *L. bonariensis* adult, previously weighed (Wi = initial weight), was placed at the center, and its feeding performance was monitored for 4 days under room temperature and darkness. At the end of the 4-day period, the insects were weighed again (Wf = final weight), and the feeding performance was assessed similarly to the no-choice bioassay (as described in Section 2.6) (n = 20).

### 2.8. Artificial Diet Tests

The feeding performance of *L. bonariensis* adults exposed to peramine was assessed through a no-choice test using an artificial diet, both following the method outlined by Faccoli and Schlyter [27]. Approximately 500 µL (density = 1.05 g mL^−1^) of the diet, comprising 87.6% water, 4.3% starch, 3.5% agar, 2.6% glucose, and 2% cellulose, was added to an Eppendorf tube (10 mm in diameter × 40 mm in length). Peramine, dissolved in water (a stock solution of 1000 ppm was prepared, from which the working concentrations indicated were obtained), was incorporated into the artificial diet at four different concentrations (45, 90, 135, and 180 ng g^−1^) and thoroughly mixed to ensure a homogeneous solution. An artificial diet supplemented with water served as the control. *L. bonariensis* was allowed to feed for 4 days in darkness at room temperature. The evaluation of feeding performance followed the procedure described earlier (see Section 2.5). Ten replicates were conducted for each of the seven experimental lines and two cultivars.

### 2.9. Data Analysis

The statistical software Statistix 10 (Tallahassee, FL, USA) was employed for data analysis. Both the *L. bonariensis* no-choice bioassays and alkaloid extract bioassays were subjected to ANOVA tests (*p* ≤ 0.05), and any statistical differences among the groups were determined using Tukey tests. Furthermore, Kruskal–Wallis tests followed by Conover–Inman tests were conducted to analyze the antifeedant effect of peramine on weevils (*p* ≤ 0.05). All percentage data were transformed to arcsine square-root. The results were presented as means along with their corresponding standard errors.

## 3. Results

### 3.1. Endophyte Infection and Quantification of Peramine

The evaluation of endophyte infection in the *L. perenne* plants is presented in Table 1, revealing significant differences in endophyte levels among the treatments using ANOVA (*p* ≤ 0.05). Among the experimental lines, LE166 exhibited the highest level of endophyte fungus infection at 100%, while LE164 showed an 85% infection rate. Additionally, the experimental lines LE162 and LE167, both exhibiting an 80% endophyte infection, shared the same level as the commercial cultivar ALTO AR1. Notably, endophyte fungus infection was not detected in the experimental lines LE165 and LE161, as well as in the commercial cultivar JUMBO. Furthermore, HPLC analysis of the alkaloidal extract from *L. perenne* leaves revealed the presence of the alkaloid peramine in the extracts from the experimental lines LE164 and LE166, as well as the commercial cultivar ALTO AR1. The peramine content varied, with concentrations of 46.5 ± 1.2 ng g^−1^ in LE166, 60.8 ± 0.4 ng g^−1^ in LE164, and 184.2 ± 36.1 ng g^−1^ in ALTO AR1. In contrast, peramine concentrations were not detected in the remaining experimental lines and the JUMBO cultivar when ANOVA was performed (Table 2).

**Figure 1:**
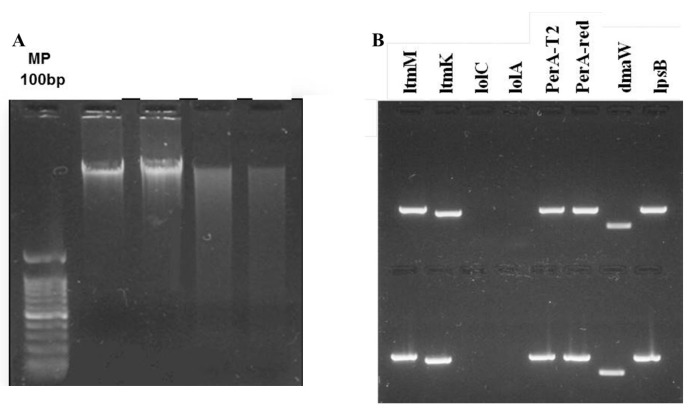
(**A**) Presence of genomic DNA from fungi belonging to the genus *Neotyphodium* sp. in representative cultivars and experimental lines of *L. perenne*. (**B**) Amplification of alkaloid genes using genomic DNA visualized through agarose gel electrophoresis of PCR.

In addition to these analyses, the sequencing of endophytic fungi in ryegrass successfully detected the presence of genomic DNA in samples from all cultivars and experimental lines, except for JUMBO (endophyte-free control), and the experimental lines LE161 and LE165. This genomic DNA belonged to the Clavicipitaceae genus and the *Neotyphodium* family (Sequence 1). Furthermore, these analyses demonstrated that the cultivars and experimental lines infected with Neotyphodium carry a gene for the expression of the alkaloid peramine (Figure 1).

**Sequence 1:** DNA sequence representative of all cultivars and experimental lines associated with endophytic infection. 

GAATGCGGTATTCGAGAACTGTAGCTGACCTGTTTCTTTCCCTCTTTTCCCCTCTAGGTTCATCTTCAAACCGGTCAGTGCGTAAGTGACAAATTCGCCGACCTCGAACGACAGGCACAAACAGCATGAAAAACTCACATTCATTTGGGCAGGGTAACCAAATTGGTGCTGCTTTCTGGCAGACCATCTCTGGCGAGCACGGCCTCGACAGCAATGGTGTGTACAATGGTACCTCCGAGCTCCAGCTCGAGCGTATGAGTGTCTACTTCAA

Definition: *Neotyphodium* sp.

Organism: Eukaryota; Fungi; Dikarya; Ascomycota; Pezizomycotina; Sordariomycetes; Hypocreomycetidae; Hypocreales; Clavicipitaceae; Neotyphodium.

### 3.2. No-Choice Bioassay

**Figure 2:**
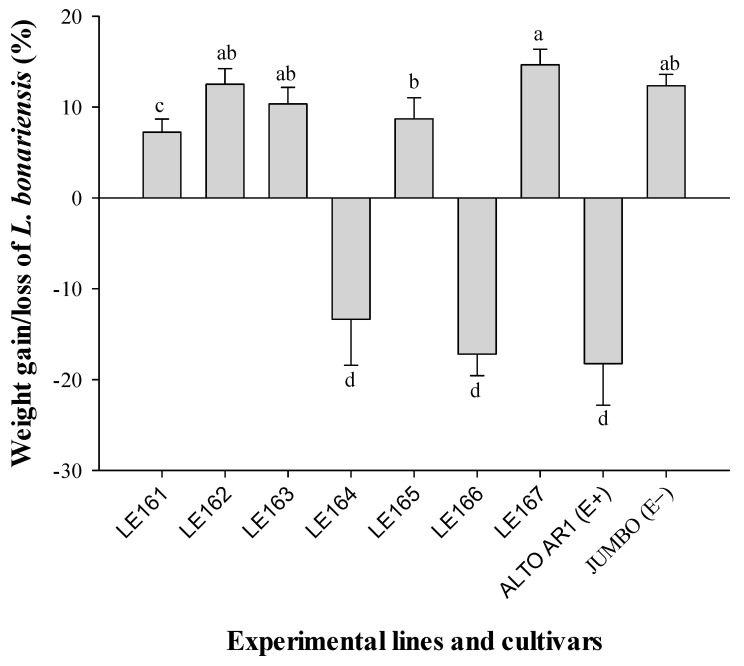
Mean weight gain, or loss (±SE), of adult *Listronotus bonariensis* in a no-choice bioassay for the seven experimental lines and two commercial cultivars of perennial ryegrass. Different letters indicate significant differences (*p* ≤ 0.05) based on ANOVA test followed by Tukey test.

The treatments exhibited a significant impact on the weight gain of *L. bonariensis*, as depicted in Figure 2. The concentration of peramine resulting from endophyte infection appeared to correlate with a substantial reduction in the weight gain of the Argentine stem weevil when ANOVA was used. Notably, in the case of the commercial cultivar ALTO AR1 (−18.2% ± 4.5) and the experimental lines LE164 (−13.3% ± 5.0) and LE166 (−17.1% ± 2.3), a notable decrease in the weight of *L. bonariensis* by 108.1%, 139.0%, and 147.9%, respectively, was observed compared to the endophyte-free perennial ryegrass cultivar JUMBO (12.3% ± 1.2). On the other hand, all remaining experimental lines, including JUMBO, which showed no production or detection of peramine due to endophyte infection, led to an increase in the weight of the Argentine stem weevil.

### 3.3. Alkaloid Extract Bioassays

**Figure 3:**
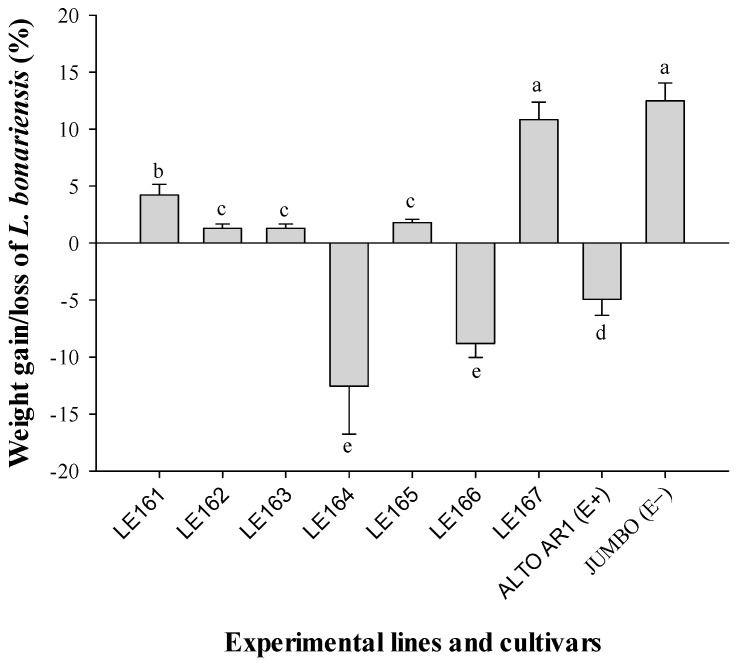
Mean weight gain, or loss (±SE), of adult *Listronotus bonariensis* fed on JUMBO leaves treated with alkaloid extracts from the seven experimental lines and two commercial cultivars of perennial ryegrass. Different letters indicate significant differences (*p* ≤ 0.05) based on ANOVA test followed by Tukey test.

When leaves of the commercial cultivar JUMBO were inoculated with alkaloid extracts from the seven different experimental lines and cultivars, significant differences (*p* ≤ 0.05) in the weight gain of *L. bonariensis* were observed when ANOVA was used (Figure 3). Notably, the experimental lines LE164 (−12.5% ± 4.1) and LE166 (−8.8% ± 1.2), as well as the commercial cultivar ALTO AR1 (−4.9% ± 1.4), exhibited a reduction in the weight gain of the Argentine stem weevil by 100.8%, 70.96%, and 39.5%, respectively, when compared to the cultivar JUMBO (12.4% ± 1.5) set as 100%. Consequently, the alkaloidal extracts from the experimental lines LE164 and LE166, as well as the cultivar ALTO AR1, demonstrated an antifeedant effect on *L. bonariensis*, leading to a significant reduction in their weight gain. Additionally, the results indicate that alkaloid extracts containing identified peramine elicited a substantial decrease in the weight gain of the Argentine stem weevil.

### 3.4. Artificial Diet Bioassays

**Figure 4:**
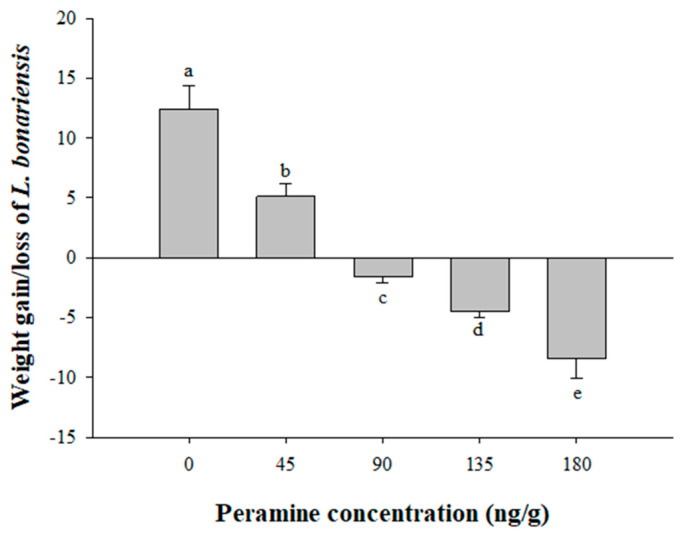
Mean weight gain, or loss (±SE), of *Listronotus bonariensis* adults evaluated in a no-choice test using artificial diet supplemented with peramine at four concentrations (n = 10). Different letters indicate significant differences (*p* ≤ 0.05) based on Kruskal–Wallis test followed by Conover–Inman test.

Figure 4 illustrates the weight gain of *L. bonariensis* when provided a diet supplemented with peramine. The incorporation of this alkaloid into the diet proved effective in inhibiting the feeding performance of *L. bonariensis* at all four evaluated doses when Kruskall–Wallis was used. The feeding performance of the Argentine stem weevil was reduced at concentrations of 45 ng g^−1^ (5.1% ± 1.0), representing a reduction of 41.1%. At 90 ng g^−1^ (−1.6% ± 0.5), the reduction was 12.9%, while at 135 ng g^−1^ (−4.5% ± 0.5), it was 36.2%. Furthermore, at 180 ng g^−1^ (−8.4% ± 1.7), the reduction reached 67.7% compared to the control treatment (12.4% ± 2.0 ng g^−1^) without the addition of peramine.

## 4. Discussion

Our data indicated that an endophytic infection of over 80% significantly reduced the feeding behavior of *L. bonariensis* (Figure 2). Several authors have reported some resistance elicited by different levels of fungal endophytes from the genus *Neotyphodium* against insect pests of various orders, including Lepidoptera, Hemiptera, Coleoptera, and Diptera [27,28,29,30,31]. This observation is consistent with the findings by Rudgers and Clay [32], who noted that endophyte-mediated herbivore resistance in *L. perenne* and tall fescue has been observed for insect species belonging to ten families and five orders. Raman et al. [33] discussed the interaction between endophytes, plants, and insects, suggesting that insects feeding on the meristematic region or leaf sheaths of the plant are mainly affected by the presence of the fungus. This applies to *L. bonariensis*, which feeds during a portion of its lifecycle on these plant parts and exhibits responsiveness to the presence of the endophyte. However, the responsiveness of herbivores to endophytes is not always correlated with the concentration of alkaloids. Krauss et al. [34] demonstrated that endophyte-infected *L. perenne* plants had no significant effect on three important aphid pests, namely *Sitobion avenae*, *Rhopalosiphum padi*, and *Metopolophium festucae* (Hemiptera: Aphididae). These authors suggested that the minimal impact of the endophyte on aphids might be attributed to the low concentrations of peramine found in ryegrass plants (ranging from 55 to 80 ng g^−1^). In contrast, our results revealed that the experimental lines LE166 and LE164 exhibited a significant antifeedant effect on the weevil when feeding performance was assessed in ryegrass leaves with a peramine content ranging between 45 and 61 ng g^−1^ (Figure 2 and Figure 3).

Antifeedant effects are not the only effects which have been reported for alkaloid extracts from forage. For instance, Dougherthy et al. [35] demonstrated that alkaloidal extracts from seed fescue infected with *N. coenophialum* exhibited a larvicidal effect against the horn fly *Haematobia irritans* (Diptera: Muscidae) and other insects when incorporated in cattle dung. Subsequently, Parra et al. [25] determined that cattle dung supplemented with the alkaloid fraction extracted from the endophytic fungi of tall fescue demonstrated significant larval mortality in *H. irritans*. HPLC analysis confirmed the presence of peramine in fecal matter. However, while our results on the presence of peramine in *L. perenne* samples demonstrated an antifeedant effect on the feeding performance of *L. bonariensis*, the alkaloid content differed from other reports. The range determined in this study was between 46.5 to 184.2 ng g^−1^ (Table 2), contrasting with values reported by Ball et al. [36] and Koulman et al. [37], who determined values ranging from 535 to 608 ng g^−1^ and 600 to 17,700 ng g^−1^ in endophyte-infected ryegrass, respectively. Additionally, Fuchs et al. [38] investigated whether the alkaloids produced by the fungus-grass association could be assimilated by plant sap-sucking aphids, finding concentrations of peramine of 23,770 ng g^−1^ in *L. perenne* plants and 18,060 ng g^−1^ in aphids, values higher than those reported in our research. In another study, Hennessy et al. [7] attributed an antifeedant effect on the lepidopteran porina larvae (*Wiseana* spp.) to the content of epoxy-janthitrem (30,600 to 83,900 ng g^−1^) in an endophyte-infected perennial ryegrass (cv. Grasslands Samson). The main difference between the concentrations determined in this study and those reported in other investigations corresponds to the age of the plants (3 months versus > 1 year, respectively). This is based on the fact that the accumulation of alkaloids tends to increase with age, reaching the highest value in 1-year-old plants [39]. This was supported by Fuchs et al. [40], who reported that the concentration of alkaloids in 1-year-old plants of *Epichloë festucae* var*. lolii* increased with plant age and even exceeded toxic levels for livestock in the summer. The observation that the alkaloidal extracts of the experimental lines LE162, LE165, and LE163 decreased the weight gain of the insects could be attributed to the production of other types of alkaloids by these endophytes, such as lolitrem B, ergovaline, or janthitrem, which could indirectly affect their feeding performance. Numerous authors have described peramine as a potent feeding deterrent against both larvae and adults of *L. bonariensis* [41,42,43,44]. According to Rowan et al. [42], concentrations of peramine lower than 10 ng g^−1^ showed no effect on *L. bonariensis*. However, concentrations ranging from 100 to 1000 ng g^−1^ demonstrated a significant decrease in feeding on an agar diet for adult weevils. The fact that certain experimental lines, such as LE166 and LE164, elicited an antifeedant effect on *L. bonariensis* adults highlights the susceptibility of these insects to peramine. Our evaluation of peramine in an artificial diet (Figure 4) contradicts reports suggesting that high concentrations are necessary to elicit an herbivorous response to this alkaloid. For example, Ball et al. [36] reported inconsistent results in a feeding bioassay with *Spodoptera frugiperda* (Lepidoptera: Noctuidae) on an artificial diet supplemented with peramine at 50,000 ng g^−1^, which showed a significant weight gain. However, doses of 10,000, 30,000, and 100,000 ng g^−1^ did not produce any discernible effect on the weight gain of the noctuid.

The significance and role of peramine as an antifeedant in herbivores have not only been demonstrated through feeding bioassays but also via molecular investigations. This aspect was discussed comprehensively in a notable review by Panaccione et al. [16], where the authors outlined the importance of bioactive alkaloids in vertically transmitted fungal endophytes. Previous studies revealed that the gene responsible for peramine biosynthesis can be rendered inactive through knockout, as noted in a feeding study involving *L. bonariensis* and ryegrass. Four treatments were assessed: Nui5 perennial ryegrass (E+), two perennial ryegrass variants with peramine biosynthesis blocked via knockout, and endophyte-free perennial ryegrass (E−). Plants lacking peramine were found to be susceptible to feeding by the curculionids, while the insects refrained from feeding on the Nui5 cultivar (E+), underscoring the impact of peramine on *L. bonariensis* susceptibility [45]. In light of this, the reduction in weight gain observed in *L. bonariensis* due to the presence of peramine in the experimental lines LE166 and LE164 indicates their potential as cultivars of ryegrass. Subsequent research will emphasize the agronomic evaluation of these two potential cultivars, their peramine production, and their influence on *L. bonariensis* under field conditions. However, an overview of our results leads us to consider the fact that peramine is not the only alkaloid that could be involved in the insect’s performance. Other alkaloid families not evaluated in this study could influence its behavior and feeding response, particularly alkaloids such as lolines. Ultimately, this information will facilitate a better comprehension of peramine’s role in the feeding behavior of *L. bonariensis* and aid researchers and managers in mitigating *L. bonariensis* infestations in South American pastures.

## Figures and Tables

**Table 1:** Mean percentage (±SE) of tillers infected with endophyte across seven experimental lines and two cultivars of *L. perenne* (n = 120).

Line Experimental	N° of Plants Tested	(E+)	(E−)	Endophyte (%) *
LE166	120	120	0	100 ± 0.0 ^a^
LE164	120	102	18	85 ± 0.8 ^b^
LE167	120	96	24	80 ± 1.1 ^b^
LE162	120	96	24	80 ± 1.1 ^b^
Alto AR1 (E+)	120	96	24	80 ± 1.1 ^b^
LE163	120	89	31	74 ± 2.5 ^c^
LE165	120	0	120	0 ± 0.0 ^d^
LE161	120	0	120	0 ± 0.0 ^d^
Jumbo (E−)	120	0	120	0 ± 0.0 ^d^

* Different letters indicated significant differences according to ANOVA followed by Tukey test (*p* < 0.05).

**Table 2:** Peramine concentration in plant tissues of six experimental lines and two cultivars of *L. perenne*.

Line Experimental	Peramine (ng/g DM) *
Alto AR1 (E+)	184.2 ± 36.1 ^a^
LE164	60.8 ± 0.4 ^b^
LE166	46.5 ± 1.2 ^c^
LE162	nd
LE161	nd
LE163	nd
LE165	nd
LE167	nd
Jumbo (E−)	nd

* Different letters indicated significant differences ANOVA followed by Tukey test according to (*p* < 0.05).

## Data Availability

The data presented in this study are available on request from the corresponding author. The data are not publicly available due to privacy.
